# Interprofessional educator development: Build it and they will come

**DOI:** 10.1007/s40037-018-0418-9

**Published:** 2018-04-16

**Authors:** Lily C. Pien, Michaela Stiber, Allison Prelosky, Colleen Y. Colbert

**Affiliations:** 1 Cleveland Clinic, Cleveland Clinic Lerner College of Medicine of Case Western Reserve University, Cleveland, OH USA; 2 Department of Allergy and Clinical Immunology, Cleveland Clinic, Cleveland, OH USA

**Keywords:** Faculty development, Educator development, Professional development, Interprofessional, Program development

## Abstract

**Background:**

We describe an interprofessional educator development program, designed intentionally, that was implemented at an academic healthcare centre. In 2014, we purposefully adapted our pre-existing educator development program to be able to include all interprofessional educators at our institution. The program’s goals were to enhance educator skills, a common need due to requirements of accreditation, and to create a local interprofessional community of teachers. The framework of the program was based upon adult learning principles, reflective practice, experiential learning and peer groups, all key characteristics of faculty development programs. It was also longitudinal and immersive. Kirkpatrick’s program evaluation model was used for identifying results; participants’ self-reported evaluation forms were collected and their narrative comments were analyzed.

**Results:**

After we opened our educator program to all interprofessional staff, our number of program participants increased. The interprofessional participants included, but was not limited to, physicians, physician trainees, nurses, physician assistants, audiologists, perfusionists, and basic science researchers. Our number of program sessions and program faculty were expanded. Our interprofessional participants reported that they were able to learn essential knowledge, skills and attitudes for their growth and development as educators, in the context of an interprofessional community, while also appreciating the diversity of their peers.

**Discussion:**

We share our insights with the redesign and implementation of an interprofessional educator program so that others can learn from our experiences. Key takeaways include using a conceptual framework for teaching effectiveness, involving interprofessional stakeholders and obtaining their perspectives, reviewing interprofessional literature and competencies, and highlighting best practices across the disciplines.

## Introduction

Professional development in education is increasingly a requirement across health professions education programs, as accreditation bodies for undergraduate and postgraduate medical education programs, nursing schools, and a variety of health professions education training programs require instructors to be trained in aspects of teaching and assessment [1–3]. In undergraduate and graduate medical education, programs can satisfy these requirements by offering stand-alone workshops or by sending core educators to national/international conferences or intensive courses. These courses may be ideal for some faculty, but can be expensive or unfeasible for institutions intent on educating a large number of their faculty, due to the operational costs associated with participant time away [4]. In addition, while healthcare is considered to be a ‘team’ sport, where interprofessional skills are viewed as essential [5], professional development programs focusing on ‘educator’ skills are not typically designed for interprofessional audiences. We describe one institution’s model for providing educator development to participants from a wide range of health professions disciplines, where the overarching goal was to enhance participants’ teaching and assessment skills. We did this to address common needs for professional development in education, meet challenges with accreditation requirements, all in the context of limited resources at the program level.

## Background

### Interprofessional education

Interprofessional education has been defined as two or more healthcare professions learning ‘from, with and about each other to improve collaboration and the quality of care’ [6]. At the postgraduate medical education level, interprofessional skills are incorporated into competencies such as systems-based practice and interpersonal and communication skills (Accreditation Council for Graduate Medical Education 2017) and the collaborator role (CanMeds 2017). Interprofessional collaboration and teamwork skills are considered to be foundational to improved outcomes in patient safety and quality improvement [7, 8]. Outcomes of interprofessional education programs typically feature skills, behaviours and knowledge related to improving teamwork skills, understanding systems of care, and collaborating together to improve patient care and healthcare delivery [9].

### Professional development in education

In this paper, we define professional development in education as those activities which enhance the knowledge, skills, attitudes and behaviours of healthcare professionals in their roles as teachers and educators. While clinical content is obviously different across healthcare fields, the ‘content’ of educational programs is fairly similar. All educators must possess knowledge of and requisite skills in curriculum design, teaching, instructional design, and assessment. Familiar challenges across healthcare fields include: limited resources at the department or institution level, lack of dedicated time for both instructors and participants, and lack of recognition for participants seeking to improve their competency in teaching [4]. Steinert’s two reviews of initiatives focused upon professional development for educators excluded programs designed for interprofessional faculty, such as instructors in nursing, nurse practitioner or allied health training programs [10, 11]. We believe the intersection of interprofessional education and educator development represents a gap in the field of professional development for educators. Innovation is required in the design and implementation of these programs due to challenges of both interprofessional education and educator development.

### Theoretical framework

Initially, we used Steinert’s model as a theoretical framework to guide program development and refinement [10, 11]. We designed a longitudinal program that incorporated adult learning principles, experiential learning, peer groups, and reflective practice. For program evaluation, Kirkpatrick’s evaluation model was utilized and focused on level 1 (reaction to the individual sessions) and levels 2a (learning as in change of attitudes) and 2b (learning as knowledge or skills acquisition) [12].

## Methods

### Program development

The *Essentials Program* was initially designed in 2005 to meet the professional development needs of faculty and instructors who taught physician trainees in a new medical school. In 2013–14, with new leadership, the program was redesigned to allow all health professions instructors at our institution to participate as educators. Goals for our program redesign included: meeting needs common to all health professions education programs, developing a wider community of learners [13], and creating an interprofessional educator culture. To do this, we met with key stakeholders, such as senior nursing educators, to discuss our program, learn about their needs, and invite participation (learners and faculty). To ensure representation of a range of professions, we conducted outreach to multiple health professions programs at our institution, met with program leadership, and then revised sessions to better reflect the roles health professions educators play, commonalities across programs, and authentic workplace settings for our diverse trainees. We also researched competency and other accreditation requirements for a variety of health professions programs and revised sessions’ activities, slides, and handouts to better meet the needs of our interprofessional audience.

Key features of the current *Essentials Program for Health Professions Educators* include: adherence to adult learning principles and reflective practice, a longitudinal curriculum [14], use of interactive instructional methods, and program evaluation based upon Kirkpatrick’s model [12]. The 10-month *Essentials* program includes a 3-hour mini retreat focused on educational theory and approximately 25 bi-monthly workshops (1.5–3 h in length). Topics include teaching skills, curriculum design, feedback (verbal and written), competency-based education and assessment, objective writing, clinical reasoning, and technology-based education. Sessions utilize pre-readings, offer opportunities to practise new skills and reinforce knowledge, and provide feedback to participants. While participants often move through the program as a cohort, they are able to sign up for individual sessions to meet their specific educational needs.

Participants include, but are not limited to, faculty and instructors who teach physician trainees, nursing trainees, physician assistants, audiologists, perfusionists, nutritionists, and basic science researchers. In 2015, we welcomed postgraduate medical education trainees into the program.

### Measurement instruments

Essentials workshops are assessed using a 10-item feedback form which includes 7–8 Likert-response items and 2 open-ended questions. The Likert scale is un-numbered and ranges from Strongly Disagree to Strongly Agree, with no neutral option. Participants are queried about their perceptions of the learning environment and teaching techniques, and their own learning.

## Evaluation

There has been a steady increase in the number of program participants, from 54 individuals in 2013–14 to 152 individuals in 2016–17. The number of sessions offered increased from 13 (2013–14) to 28 (2016–17). Workshop participation in 2016–17 ranged from 6 individuals per session to 41 per session, for a total annual participation count of 507 across all sessions in 2016–17 (up from 221 in 2013–14). Instructional hours increased from 25.5 h (2013–14) to 54.5 (2016–17). The number of instructors in the program grew from 8 to 16 during the same time period.

For the December 2014–17 time period (interprofessional audience), 944 evaluations were collected from 1,132 total Essentials workshop participants, for an average of 83.4% evaluation response rate. In aggregate, participant assessment of individual workshop teaching techniques ranged from 3.5 to 4.0/4.0 (mean = 3.8). Participants’ assessment of their own learning ranged from 3.35 to 4.0/4.0 (mean = 3.7). Respondents’ assessment of their own learning and perception of teaching techniques indicate that a high degree of satisfaction was maintained as the program moved to an interprofessional educator development model.

Narrative comments from feedback forms are routinely collected as part of quality improvement efforts from year to year. Comments comprised two main categories: general comments about the sessions and remarks about the interprofessional nature of the sessions. Comments were related to perceptions of new knowledge and skills, opportunities to practise, changes in attitudes, desires to use new teaching skills with learners in the workplace, and appreciation of interactive techniques (Tab. 1).

**Table 1:** Narrative comments

*Comments related to educational topics and skills*	‘Great session! Learned many techniques & apply as a teacher’
	‘I felt this was a very useful session. I can think of 2 to 3 things I can specifically use to enhance my lectures now’
	‘Helpful to practise methods through scenarios’
	‘More confident about material & will go back & rewrite our phlebotomy objectives to build better curriculum’
*Comments related to interprofessional faculty development*	‘Thank you for continuing to promote interprofessional collaboration, I hope this continues into the future’
	‘Group work valuable especially working with multidisciplinary group of educators’
	‘Working in groups is helpful to gain insight into other perspectives and disciplines’
	‘Liked incorporation of examples from different professions’
	‘Loved having an interdisciplinary group for this. I learned processes within physician practice that I never realized—probably thought about but never really clicked’

## Discussion

We have described a longitudinal model for professional development in education which is innovative in its goals, design and inclusion of interprofessional participants. To our knowledge, this program’s focus on skill development for interprofessional educators appears unique in the health professions literature. The evaluation of our program provides support for the development of local, institutional and interprofessional educator professional development programs. We believe our educator development program has been successful in accomplishing several goals: improving and enhancing teaching skills; standardizing teaching practices at the institution; and fulfilling accreditation agencies’ requirements to provide professional development in education for key instructors.

### Lessons learned

What follows is a list of lessons learned over the past four years which may be helpful to other programs as they design and implement interprofessional educator development programs.

#### Engage in reflection

When planning your program and sessions, reflect upon current stand-alone offerings of professional development for educators or existing programs with an openness for adaptation and innovation to meet the needs of diverse participants [8]. Steinert’s conceptual framework for teaching effectiveness can be used to guide program development [10, 11]. Emphasis on Steinert’s framework, including the use of experiential learning and peer groups, was appreciated by the interprofessional audience, as noted in their evaluation comments.

#### Obtain feedback from stakeholders

Obtain ongoing feedback from key stakeholders, representing your many constituencies, to help support a safe, collaborative and interprofessional learning environment. We routinely send draft cases to faculty to ensure our scenarios are authentic and representative of their learning environments.

#### Consider recruiting interprofessional faculty

By utilizing a variety of health professions educators, your program not only reflects institutional support for interprofessional interactions but also provides professional role models for participants in the program.

#### Ensure alignment at all levels

We recommend that program planners ensure that the program’s mission and values drive program development. Everything from session objectives to workshop materials to the instructors themselves should reflect the interprofessional nature of the program [8]. Even our workshop evaluations were crafted to ensure that all participants would feel their profession was represented and perspectives valued. Having a vision of an interprofessional program and planning for alignment at all levels can aid in the implementation of an interprofessional program.

#### Promote interprofessional interaction

We recommend assigning seating to ensure opportunities for interprofessional interaction during educator development sessions. This can be accomplished by attaching numbered cards (e. g., 1, 2, 3, and 4) to session handouts which participants pick up at the beginning of a session. Tables or seating areas are also numbered accordingly and participants sit at the assigned tables. We have found that colleagues from specific fields or disciplines often arrive in pairs.

#### Learn from each other

During sessions, continuously work to illustrate commonalities across fields, but also highlight best practices across fields which everyone can learn from. Lastly, be open to unplanned and unexpected outcomes [15]. For example, some participants have wanted to share materials with their discipline-specific colleagues, either by providing a scholarly article to their specialty journal or by directly teaching the materials to their peers.

### Limitations

Our experience in offering interprofessional educator development may not generalize to all settings. This description is limited to one setting, and our measures of success relied heavily on participants’ self-reports, our only outcome measure. Our sessions have elicited high engagement from participants, which may not be the case in all environments.

## Conclusion

Interprofessional educator development is critical in today’s complex healthcare environments, where there are limited resources and increasing needs for professional development of educators. We have described a longitudinal professional development program for educators that aims to meet the needs of a wide spectrum of adult learners. We speculate that the success of this program may be related to considerations of program planning, engagement of stakeholders, use of feedback to improve sessions, and workshop topics (e. g., providing feedback, competency-based education and assessment), which may have had significant appeal for our interprofessional audience. We believe that the development of programs like this is an evolving area, and lessons learned within our context may be helpful to other program developers as they design and implement their own programs.
